# Vitamin D supplementation improves serum markers associated with hepatic fibrogenesis in chronic hepatitis C patients: A randomized, double-blind, placebo-controlled study

**DOI:** 10.1038/s41598-017-09512-7

**Published:** 2017-08-21

**Authors:** Piyawat Komolmit, Sayamon Kimtrakool, Sirinporn Suksawatamnuay, Kessarin Thanapirom, Kanita Chattrasophon, Panarat Thaimai, Chintana Chirathaworn, Yong Poovorawan

**Affiliations:** 1 Division of Gastroenterology, Department of Medicine, Faculty of Medicine, Chulalongkorn University, Bangkok, Thailand; 2 Center of Excellence in Liver Diseases: King Chulalongkorn Memorial Hospital, Thai Red Cross Society, Bangkok, Thailand; 3 Gastroenterology Unit, Department of Medicine, Lerdsin Hospital, Bangkok, Thailand; 4 Gastroenterology Unit, Department of Medicine, Taksin Hospital, Bangkok, Thailand; 5 Division of Immunology, Department of Microbiology, Faculty of Medicine, Chulalongkorn University, Bangkok, Thailand; 6 Center of Excellence in Clinical Virology, Faculty of Medicine, Chulalongkorn University, Bangkok, Thailand

## Abstract

Hepatic fibrosis is the net accumulation of matrix tissue components which controlled by pro-fibrolytic enzymes, matrix metalloproteinases (MMPs), and pro-fibrotic cytokine, TGF-β_1_, and enzymes, tissue inhibitors of MMPs (TIMPs). Vitamin D (VD) supplementation has been shown to reverse these processes *in vitro* and *in vivo*. This study sought to determine the effect of VD supplementation on serum fibrotic markers in chronic hepatitis C (CHC) patients. Fifty-four CHC patients with VD deficiency were randomized into two groups, a VD group (n = 29) and a placebo group (n = 29). The serum levels of 25-hydroxy VD, TGF-β_1_, TIMP-1, MMP2 and MMP9 were measured at baseline and at the end of the 6-week study period. Upon correction of VD levels, TGF-β_1_ and TIMP-1 levels were decreased, and the MMP2 and MMP9 levels were significantly increased in the VD group. A comparison of the mean changes (delta) in the markers between groups showed that TGF-β_1_ and TIMP-1 levels were significantly decreased and the MMP2 and MMP9 were significantly higher in the VD group than in the placebo group. By using CHC patients as a model, this study provides additional evidence that VD plays an important role in the reversal of hepatic fibrogenesis.

## Introduction

Liver fibrogenesis is a dynamic healing process initiated in response to hepatic injury and inflammation. This process stimulates liver fibroblast-like cells, hepatic stellate cells (HSCs) and portal myofibroblasts, resulting in the accumulation of extracellular matrix (ECM) components in the liver parenchyma and ultimately progressing to severe cirrhosis, the disruption of vascular circulation and liver cell dysfunction^1^. The imbalance in pro-fibrotic cytokines/enzymes, e.g., transforming growth factor beta 1 (TGF-β1) and tissue inhibitors of matrix metalloproteinases (TIMPs), and antifibrotic enzymes, e.g., matrix metalloproteinases (MMPs), plays an important role in the local control of progressive or reversible scarring^2^.

Chronic viral hepatitis C (CHC) is currently among the leading causes of the global burden of disease, causing morbidity and mortality from cirrhosis and hepatocellular carcinoma^3^. Vitamin D (VD) deficiency is common among patients with chronic liver diseases regardless of the etiology, and the degree of deficiency is correlated with the severity of liver dysfunctions^4–6^. Over 70 percent of CHC patients show some degree of VD deficiency^7^. CHC genotype 1 patients with VD deficiency show lower responses to standard pegylated interferon/ribavirin treatment, while the response rate in difficult-to-treat patients increases after the restoration of VD levels^7, 8^. Clinical data also suggest that CHC patients with VD deficiency have more severe progressive liver fibrosis and liver stiffness, leading to the hypothesis that VD might be involved in liver fibrogenesis^9, 10^.

VD is currently known to have a wide variety of non-calcium homeostasis functions; the active form, 1,25-hydroxyvitamin D3 [1,25(OH)VD], can be generated locally in several tissues and exerts para or autocrine functions via the vitamin D receptor (VDR). VD is involved in immune cell regulation and proliferation and maintains balanced innate and adaptive immune responses^11^. Activated HSCs, in response to the fibrogenic cytokine TGF-β1, upregulate the expression of *VDR* mRNA^1, 12^. *VDR* knockout mice spontaneously develop cirrhosis^13^, and *VDR* and *DHCR7* gene polymorphisms are associated with more severe liver fibrosis in CHC patients^14, 15^. Furthermore, a non-calcemic VD derivative, calcipotriol, was shown to prevent liver fibrosis in a mouse model of cirrhosis^13, 16^.

Convincing evidence from *in vitro* and animal models has demonstrated that VD is involved in hepatic fibrogenesis. Thus far, at the clinical level, the concept that low VD may influence and be related to the severity of liver fibrosis is supported by clinical observations based on histological evidence, liver stiffness measurements and the association with VD/VDR metabolic pathway polymorphisms. However, some discrepant data have suggested otherwise^17, 18^. Several factors such as genetics, food intake, ultraviolent B (UVB) radiation exposure and stage of liver disease affect VD status, and many factors such as the disease etiology, time course and treatment influence liver fibrosis. VD deficiency alone might contribute in some part to hepatic fibrogenesis, and its influence might be modified by other factors.

However, there is currently no clinical evidence that VD supplements can delay or prevent the progression of liver fibrosis^19^. To support this hypothesis, we conducted a randomized double-blinded, placebo-controlled trial to assess the dynamic changes in serum fibrogenic cytokines/enzymes in CHC patients with VD deficiency after short-term supplementation with VD.

## Results

Between February and December 2014, 73 CHC patients were assessed for eligibility; of them, 14 patients were excluded due to 25(OH)VD levels of ≥30 ng/mL, and 1 CHC patient was excluded for not providing written informed consent. A total of 58 CHC patients were included in this study and were randomly assigned to the VD group (n = 29) or the placebo group (n = 29).

Patient age ranged from 26 to 70 years old with a mean age of 50.3 ± 10.6 years. There were 36 male patients (62.1%) and 22 female patients (37.9%). Fifty patients were naïve cases (86.2%), and 8 patients were relapsers (13.8%). Twenty-one patients were diagnosed with compensated liver cirrhosis (36.2%). The mean HCV-RNA level was 5.59 ± 0.82 log IU/mL. A total of 31 and 27 patients had HCV genotype 1 and non-genotype 1, respectively.

As shown in Table 1, at baseline, there were no significant differences in the demographic, clinical or biochemical patient data between the groups. The proportion of male patients was higher in the VD group than in the placebo group, but this difference was not significant (p = 0.10). The fourteen cases of non-genotype 1 HCV in the VD group included twelve cases of genotype 3, one case of genotype 2 and one case of genotype 6. All thirteen cases of non-genotype 1 HCV in the placebo group were composed of genotype 3. During the 6-week follow-up period, no adverse events were reported.

**Table 1:** Demographic, clinical and laboratory data at baseline in the VD and placebo supplementation groups.

	VD supplementation (n = 29)	Placebo (n = 29)	p - value
Age (years)	50 ± 11.7	50.4 ± 9.5	0.84
Gender: Male, n (%)	21 (72.4)	15 (51.7)	0.10
Body mass index (kg/m^2^)	24.8 ± 3.8	24.6 ± 3.0	0.54
Cirrhosis, n (%)	11 (37.9)	10 (34.5)	0.78
Naïve cases, n (%)	26 (89.6)	24 (82.7)	0.45
Liver stiffness (kPa)	16.2 ± 10.8	15.0 ± 7.8	0.62
Patients with significant fibrosis (≥ F2), n (%)	23 (79.3)	25 (86.2)	0.49
HCV-RNA (log IU/mL)	5.66 ± 0.84	5.52 ± 0.80	0.81
HCV viral load ≥log 6 IU/mL, n (%)	10 (34.5)	7 (24.1)	0.32
HCV genotype - Genotype 1, n (%) - Non-genotype 1, n (%)	15 (51.7) 14 (48.3)	16 (55.2) 13 (44.8)	0.79
SGOT (U/L)	116.4 ± 115.2	87.0 ± 63.3	0.24
SGPT (U/L)	119.3 ± 108.4	115.4 ± 127.2	0.89
25(OH)VD (ng/mL)	19.9 ± 5.3	19.0 ± 5.4	0.53
TGF-β_1_ (pg/mL)	661.3 ± 214.6	609.5 ± 275.9	0.42
TIMP-1 (ng/mL)	2.3 ± 0.6	2.0 ± 0.6	0.10
MMP2 (ng/mL)	416.5 ± 136.9	441.1 ± 162.3	0.50
MMP9 (ng/mL)	179.8 ± 100.5	158.6 ± 93.5	0.40

### Changes in VD levels

**Figure 1:**
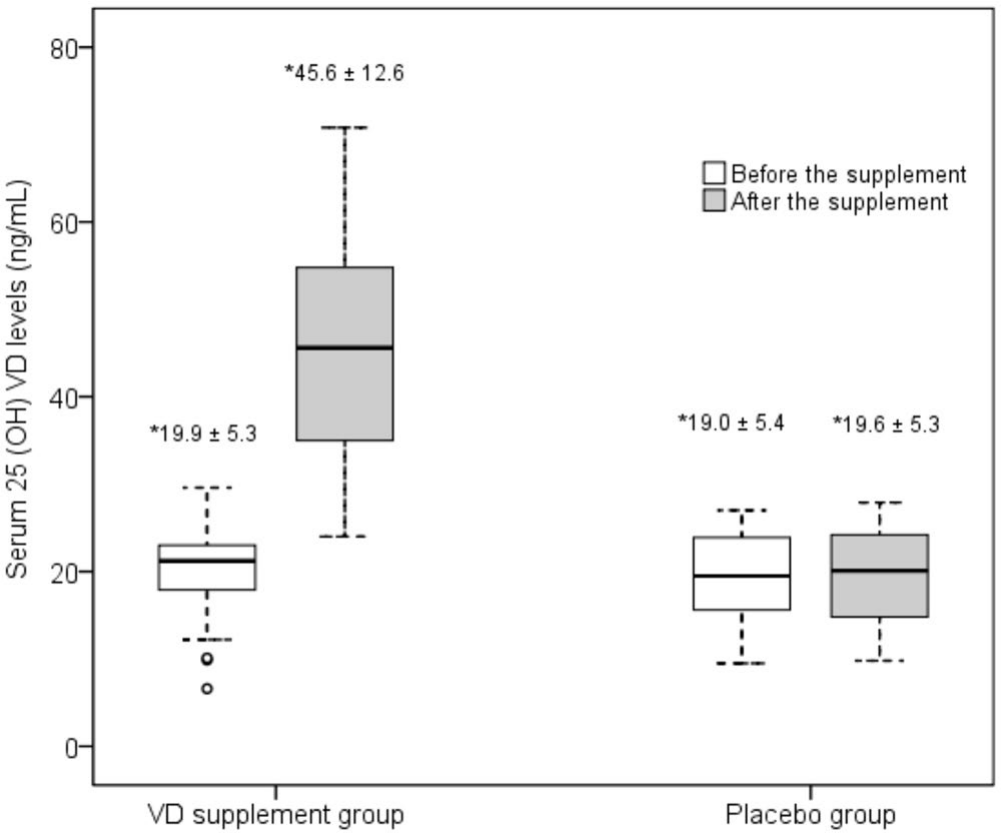
Box plot of serum 25(OH)VD at baseline and after VD or placebo supplementation for 6 weeks. White and gray boxes represent median and interquartile ranges at baseline and 6 weeks, respectively. *Numbers that indicate mean ± SD are shown at the top of each time point.

In the VD group, the mean serum 25(OH)VD levels significantly increased to normal levels compared to the baseline level (45.6 ± 13.1 vs. 19.5 ± 5.3 ng/mL, p < 0.001). In the placebo group, there was no change in the mean serum 25(OH)VD level during the 6-week follow-up period (18.6 ± 5.1 vs. 18.2 ± 5.5 ng/mL, p = 0.3) (Fig. 1). The detail summary of VD levels in each group according to severity of vitamin D levels and the changes of VD levels at 6-week follow up were submitted in Supplementary Table S1. In both the VD and placebo groups, patients with VD levels below 20 ng/mL (VD deficiency) or from 20 to less than 30 ng/mL (VD insufficiency) at baseline showed the same changes in VD levels after supplementation for 6 weeks as the patients with VD levels below 30 ng/mL (Supplementary Table S2).

In the VD group, 3 of the 29 patients had post-VD replacement levels below 30 ng/mL. In one of these patients, the initial VD level was slightly low at 29.6 ng/mL, and after VD replacement, the level was 27.9 ng/mL (a reduction of 1.7 ng/mL). For the other two patients, the initial VD levels indicated deficient statuses (below 20 ng/mL), and after VD replacement, the levels increased by 8.4 and 10.1 ng/mL, respectively, which raised their VD statuses to insufficient. Delta changes in TGF-β1, TIMP-1, MMP2 and MMP9 levels mainly indicated a decrease in fibrogenesis (Supplementary Table S3). As there were only 3 patients, no specific factors related to this phenotype could be demonstrated. In the placebo group, VD levels remained low in all patients.

Assessing adherence to the prescribed medications was performed using pill counts and patient interviews at the end of 6-week follow up. There were two cases (2/58 cases; 3.5%), one case in each group, who missed a single dose of the medications. By objective measurement, all but one patient in VD group had increased VD levels at the end of follow up. The patient who had reduced VD level (1.7 ng/mL) had good compliance and completed all doses of VD supplement.

### Changes in the levels of serum markers after the 6-week period in each group

The level of TGF-β1 in the VD group decreased from 661.3 ± 5.3 to 636.7 ± 213.1 pg/mL; this decrease was not statistically significant (p = 0.2). However, the mean level of TGF-β1 in the placebo group significantly increased during the same period from 609.5 ± 275.9 to 666.6 ± 282.3 pg/mL (p = 0.008) (Table 2).

**Table 2:** Mean serum levels of each marker at baseline (Pre) and after 6 weeks of follow-up (Post) in the VD and placebo groups.

Serum marker (ng/mL)	VD supplementation group (n = 27)			Placebo group (n = 27)		
	Pre- mean ± SD	Post- mean ± SD	p-value	Pre- mean ± SD	Post- mean ± SD	p-value
25(OH)VD	19.9 ± 5.3	45.6 ± 12.6	<0.001	19.0 ± 5.4	19.6 ± 5.3	0.2
TGF-β_1_*	661.3 ± 214.6	636.7 ± 213.1	0.2	609.5 ± 275.9	666.6 ± 282.3	0.008
TIMP-1	2.3 ± 0.6	2.1 ± 0.6	0.1	2.0 ± 0.6	2.4 ± 0.7	0.01
MMP2	399.6 ± 141.0	451.3 ± 157.7	0.05	420.4 ± 138.8	378.5 ± 109.9	0.1
MMP9	176.6 ± 91.9	210.8 ± 89.6	<0.001	158.5 ± 95.4	135.2 ± 80.1	<0.001

*pg/mL.

The changes in mean TIMP-1 levels in both groups were similar to the changes in TGF-β1. The mean serum TIMP-1 levels in the VD group were slightly decreased from 2.3 ± 0.6 to 2.1 ± 0.6 (p = 0.1). In the placebo group, the mean TIMP-1 levels significantly increased from 2.0 ± 0.6 to 2.4 ± 0.7 (p = 0.01).

The mean serum MMP2 and MMP9 levels in the VD supplementation group significantly increased from 399.6 ± 141 to 451 ± 157.7 ng/mL (p = 0.05) and from 176.6 ± 91.9 to 210.8 ± 89.6 ng/mL, respectively (p < 0.001). The mean serum MMP2 levels were not changed in the placebo group. In contrast to the VD group, the MMP9 levels in the placebo group significantly decreased from 158.5 ± 95.4 to 135.2 ± 80.1 (p < 0.001) (Table 2).

### Comparison of the mean changes (delta changes) in the serum markers

**Figure 2:**
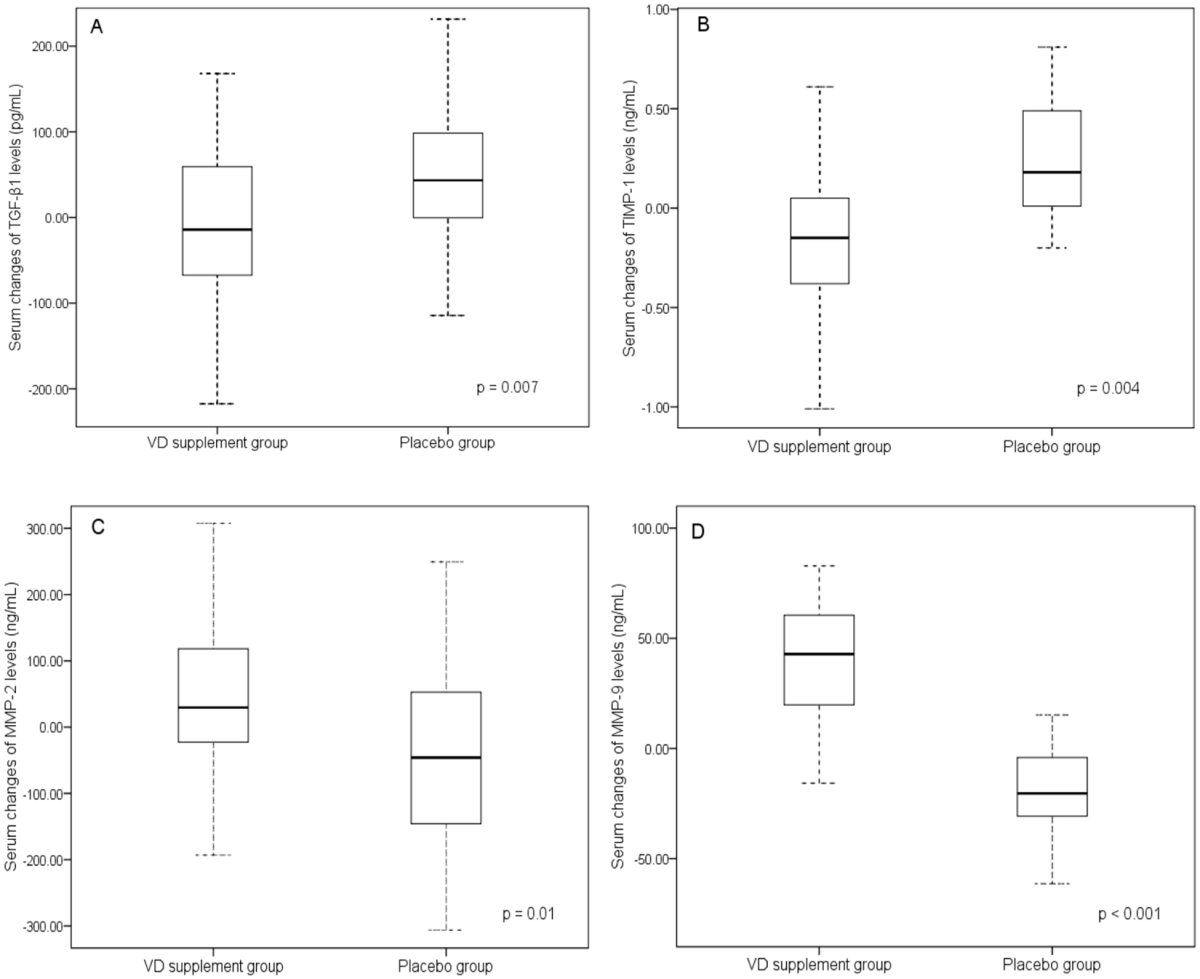
Box plot of mean differences (delta) in serum markers after 6-week supplementation with VD or placebo.

**Figure 3:**
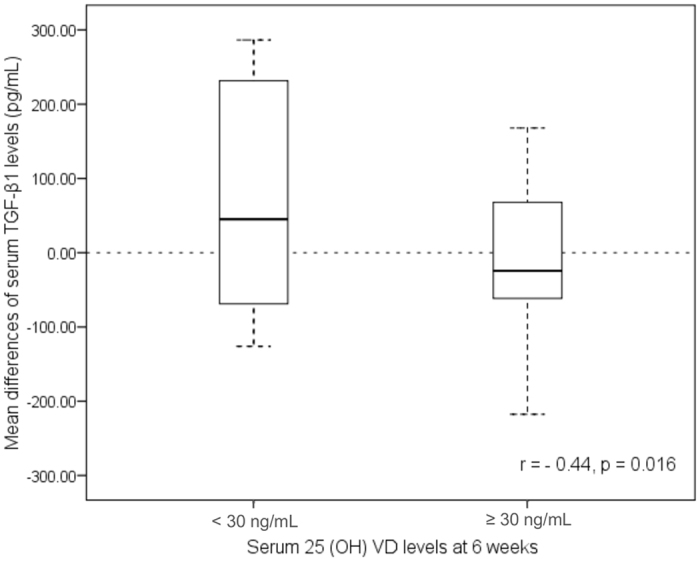
Box plot of serum 25(OH)VD levels and the mean difference in serum TGF- β1 after VD or placebo supplementation for 6 weeks.

When comparing the mean changes in 25(OH)VD and the serum markers in the groups, all markers showed significant changes concomitant with the increase in VD levels (Table 3 and Fig. 2). The mean change in 25(OH)VD was significantly greater in the VD group (25.6 ± 13.5 ng/mL) than in the placebo group (0.5 ± 2.7 ng/mL, p < 0.001). The delta changes in TGF-β1 (−24.6 ± 113.1 pg/mL) and TIMP-1 (−0.1 ± 0.4 ng/mL) were decreased in the VD group, but increased in the placebo group (57.1 ± 107.4 pg/mL and 0.3 ± 0.7 ng/mL, respectively) (Table 3 and Fig. 3). These delta changes were statistically significant (p = 0.0098 and p = 0.0129, respectively). The delta changes in mean serum MMP2 and MMP9 were the opposite of those observed for TGF-β1 and TIMP-1 (Table 3 and Fig. 2). The delta changes in mean serum MMP2 and MMP9 were increased in the VD group (51.7 121.3 and 34.2 38.5 ng/mL, respectively) but decreased in the placebo group (−41.9 ± 128.6; p = 0.0133 and −23.3 ± 26.6 ng/mL; p < 0.001, respectively). The effect size analysis suggested that VD supplementation strongly affected the change in the mean VD level, with a moderate (0.7) to large (1.74) effect on the changes in TGF-β1, TIMP-1, MMP2 and MMP9.

**Table 3:** Comparisons of changes in the groups after 6 weeks of supplementation.

Changes of serum or delta (Δ) levels (ng/mL)	Vitamin D (n = 29) Mean ± SD	Placebo (n = 29) Mean ± SD	Effect size^B^	Estimate (SE)^D^	95% CI of estimate (lower, upper)^D^	*P* values^C^
25(OH)VD	25.7 ± 13.5	0.5 ± 2.7	2.59	25.21 (2.40)	20.40, 30.03	<0.0001
TGF-β_1_^A^	−24.6 ± 113.1	57.1 ± 107.4	0.74	−79.05 (30.24)	−139.74, −18.37	0.0117
TIMP-1	−0.1 ± 0.4	0.3 ± 0.7	0.70	−0.40 (0.16)	−0.73, −0.07	0.0194
MMP2	51.7 ± 121.3	−41.9 ± 128.6	0.75	86.30 (33.80)	17.99, 154.62	0.0146
MMP9	34.2 ± 38.5	−23.3 ± 26.6	1.74	62.35 (9.13)	43.89, 80.81	<0.0001

^A^pg/mL; ^B^Cohen’s d effect size: 0.2 small, 0.5 medium, 0.8 large magnitude of effects.

^C^Comparison between two groups was performed through ANCOVA. In ANCOVA, the dependent variable is the post-test measure, and the pre-test measure was a covariate and controlled for. And also controlled were cirrhosis, gender and genotype status.

^D^Estimate is the adjusted difference between treatment and placebo group (pre-test measure was adjusted for).

A. TGF-β1. B. TIMP-1. C. MMP2. D. MMP9. Each box shows the median, 25^th^ and 75^th^ percentiles, and error bars for maximum and minimum values; p values represent significant differences in the mean differences (delta) between the VD and placebo groups.

### Correlation of 25(OH)VD levels and delta changes of TGF-β1 levels at 6 weeks

In the correlation analysis, a serum 25(OH)VD level ≥ 30 ng/mL at 6 weeks of supplementation showed a significant negative association (r = −0.44; p = 0.016) with the delta change of serum TGF-β1 levels, as shown in Fig. 3.

The dashed line denotes the zero value of the y-axis. Each box shows the median, 25^th^ and 75^th^ percentiles and error bars for maximum and minimum values. A significant negative correlation between the two parameters is shown by the r value.

### Changes in transaminase levels

Changes in transaminase levels were calculated from the paired pre-and post-supplement levels of 22 cases and 23 cases in VD and placebo groups, respectively. After 6 weeks, the patients in the VD group exhibited significant decreases in both the AST and ALT levels, as indicated by the mean ALT values (116.7 ± 96.6 vs 78.0 ± 60.3, mean change of −38.1 ± 70.5, p = 0.02, 95%CI [−69.3, −6.8]). In contrast, none of the patients in the placebo group showed significant changes in the mean AST or ALT level (Supplementary Table S4).

There was no evidence of hepatitis flares in the placebo group. Only three patients in this group presented with ALT levels above 200 IU/mL at the 6-week time point. In these patients, minimal changes from the baseline ALT levels were observed (233 to 240, 198 to 260 and 255 to 274 IU/mL). These three patients also exhibited increases in TGF-β1 levels from 412 to 464, 930 to 1114.6 and 284 to 298 pg/mL, respectively. However, several other patients in the placebo group who had a mildly elevated ALT level at baseline and presented with minimal changes in this level at the 6-week time point also presented with a TGF-β1 level above 1000 pg/mL.

### Changes in hepatitis C viral loads

HCV RNA viral loads of the plasma samples that had been stored at −80 °C and had not been previously thawed were re-evaluated. We collected paired samples pre- and post-supplementation from each arm of 27 of the 29 patients; thus, a total of 108 samples were quantitated. The baseline mean viral loads of the stored plasma samples exhibited a slight but significant decrease compared with the viral loads measured immediately at the beginning of the trial (VD 5.29 ± 1.0 vs 5.57 ± 0.83 log IU/mL, p = 0.028, 95%CI [−0.54, −0.03]) (Supplementary Table S5A).

The dependent t-test was used to compare the mean HCV viral loads between paired samples pre-and post-supplementation in both the VD and placebo supplement groups, and no significant changes were observed (5.29 ± 0.94 vs 5.3 ± 0.88 log IU/mL, p = 0.93, and 5.26 ± 1.07 vs 5.3 ± 1.15 log IU/mL, p = 0.92, respectively) (Supplementary Table S5B).

### Subgroup analysis

Subgroup analyses of the VD supplement group were performed according to gender, severity of VD deficiency, hepatitis C genotype and cirrhotic status, which revealed that there were no specific factors that had a significant effect on patient outcomes, as shown in Supplementary Table S6.

## Discussion

Chronic hepatic injury and inflammation constitute the initial step in hepatic fibrogenesis, according to clinical experience with patients with CHC. Upon activation by fibrogenic cytokines, mainly TGF-β1, quiescent HSCs are transformed into activated myofibroblast-like cells. The accumulation of fibrillar components secreted by these activated HSCs in the ECM leads to progressive fibrosis and cirrhosis. The local control of ECM accumulation depends on the balance between MMPs, which are degradation enzymes, and TIMPs, which are fibrogenic enzymes secreted mainly from activated HSCs that function as MMP inhibitors.

An increase in TGF-β1 levels and a shift in favor of TIMPs results in progressive fibrosis, while in the opposite direction, an increase in MMP functions facilitates fibrosis degradation and tissue remodeling^20^.

The results of our study showed that the restoration of VD levels to the normal range in patients with CHC who had VD deficiency resulted in a shift in the pro-fibrogenic cytokines and enzymes in favor of pro-fibrogenic degradation. In the VD group, serum TGF-β1 and TIMP-1 levels were significantly decreased and MMP2 and MMP9 levels were significantly increased compared with those in the placebo group. Importantly, these changes occurred even though CHC remained active. In other words, the main driving force of hepatic fibrosis persisted, and the additional pro-fibrotic effect of VD deficiency was likely minor, although some changes in the marker levels were still detected following VD supplementation and restoration.

The current study used the model of CHC-induced fibrogenesis, as there is substantial evidence supporting the relationship between VD deficiency and the outcomes of this disease. Clinical observations have indicated that VD deficiency is common among CHC patients and worsens with the severity of liver dysfunction and fibrotic scores^7^. CHC patients with VD deficiency also have lower sustained virologic responses (SVRs) to interferon/ribavirin therapy^8^. Another point of interest from the perspective of hepatology is the association between VD deficiency and liver fibrosis; it is thought that VD deficiency may constitute an additional factor, rather than an outcome, that contributes to more severe fibrosis, especially in CHC patients^21, 22^. Most of the data indicating that VD and VD supplementation can reverse or prevent fibrosis were obtained from *in vitro* studies and animal models.

The aim of the present study was to demonstrate and support the concept that VD supplementation might benefit CHC patients with VD deficiency due to its role in the reversion of the changes in serum fibrogenic cytokine and enzymes to a pro-fibrolytic state. Whether the restoration of VD deficiency could improve or delay fibrosis during ongoing HCV infection or treatment is difficult to evaluate via follow-up tissue biopsies in patients with CHC. These difficulties could also be seen in placebo-controlled trials of the treatment of non-alcoholic steatohepatitis (NASH) with high-dose vitamin E or ursodeoxycholic acid; these studies lasted for two years and did not show an improvement in hepatic fibrosis^23, 24^.

In addition, increasing data have confirmed the benefit of curative treatment on the improvement of liver functions and the regression of fibrosis in patients with CHC and chronic hepatitis B^25–27^, which, in turn, has diminished interest in an additional fibrotic therapy for these diseases. The new generation of direct-acting antivirals (DAAs) for CHC achieves a curative rate of over ninety percent^28^. An evaluation of the benefit of the usual dose of VD would require a thousand pairs of biopsy tissues and a long-term follow-up study of at least 5 years. In our view, the demonstration of this benefit in human subjects could be performed indirectly by assessing immunologic or enzymatic changes as surrogate markers for the reduction of fibrosis to support the results of experimental studies.

VD3 exerts its effect via the VDR, which is expressed and upregulated in activated HSCs^29^. VD3 upregulates VDR expression and suppresses TGF-β1-stimulated TIMP-1 mRNA expression and increases MMP activity in primary rat HSCs^30^. VD supplementation in rats has been shown to prevent and reduce the degree of hepatic fibrosis induced by thioacetamide^30^. VD exerts its effects via the nuclear receptors VDR and retinoid X receptor by interacting with their *cis*-regulatory elements, which control genes that are involved in hepatic fibrogenesis to reduce the pro-fibrotic effects of TGF-β1^13^.

Synthetic VD without hypercalcemic properties (calcipotriol) was shown to inhibit fibrogenesis in a mouse model of hepatic fibrosis^13^. In CCl4 rats with established fibrosis and cirrhosis, VD3 supplementation prevented the progression of fibrosis or cirrhosis, suppressed TGF-β1 expression and increased MMP9 levels in liver tissues; interestingly, no amelioration of established fibrosis was demonstrated^31^. A recent experiment that was more relevant to humans evaluated the effect of VD on isolated primary human HSCs and showed that VD could inhibit TGF-β1/SMAD signaling-induced fibrogenesis^12^. The expression of TGF-β1 and several other pro-fibrotic genes was decreased or attenuated upon VD supplementation, and these effects were observed in the presence of VDR and the non-VDR receptor PDIA3^12^. However, the dosage of VD3 used in cell culture or animal models could not be extrapolated to the optimal dosages in human trials; thus, whether the dose should be physiological or high, with the associated risk of hypercalcemia, remains unclear. Thus far, no clinical studies have evaluated VD supplementation, high-dose VD or calcipotriol in patients with chronic hepatitis or cirrhosis to support the benefits seen *in vitro* or in animal models. Our study is the first to evaluate the benefit of VD restoration on hepatic fibrogenesis in a clinical situation.

Our study used specific serum markers to represent local tissue phenomena. For instance, the serum TGF-β1 concentration is correlated with the degree of liver fibrosis in CHC patients, and serum TGF-β1 levels are reduced in CHC patients who respond to interferon therapy and are associated with the regression of liver fibrosis^12, 32^. MMPs are initially secreted by local pro-fibrogenic macrophages and activated HSCs. During fibrotic remodeling and degradation, MMPs are derived from pro-resolution, reparative macrophages that have differentiated from systemic monocytes^33^. These cells secrete MMPs, which promote matrix degradation, and express TNF-related apoptosis-inducing ligand (TRAIL) and MMP9, which induce activated HSC apoptosis^34^. Several studies have used the serum levels of MMPs and TIMPs as markers to show the changes in fibrotic stages in CHC patients. For instance, one study in CHC patients demonstrated the association between serum TIMP-1 and the METAVIR hepatic fibrosis score in pretreatment biopsies. In addition, patients who received interferon/ribavirin treatment and achieved a sustained virologic response at 24 weeks showed an increase in serum MMP9 and a decrease in serum TIMP-1 levels^35^. A study by Leroy *et al*.^36^ showed an increase in the serum concentration of MMP2 along with an increase in fibrotic stages and a decrease in serum MMP9 during fibrosis progression. The discrepancy in the changes in serum MMPs among studies in CHC patients might depend on several factors, especially the dynamic phases of the disease, as the pro-fibrogenic and pro-fibrolytic phases require different functions of MMPs. The main sources of MMP2 and MMP9 are activated HSCs and pro-resolution macrophages, respectively^33, 34^. Our study was conducted during active chronic infection without the interference of antiviral drugs or the immune modulator effects of interferon. If the driving force of the restoration of VD deficiency to physiological levels is sufficiently robust, the balance of fibrogenesis should shift to the pro-fibrinolytic stage; this shift should be reflected by reductions in TGF-β1 and TIMP-1 and increases in MMP2 and MMP9 in the serum, as demonstrated in this study.

Further, it would be interesting to investigate why TGF-β1 levels in the placebo group increased without any intervention. There were also no significant changes in transaminase levels in this group. We hypothesize that the ongoing active fibrogenesis partly contributed to the increased TGF-β1 levels. However, the continuation of active infection or inflammation might have also contributed to these increased TGF-β1 levels, as changes in transaminase levels usually but do not always reflect the magnitude of hepatic tissue inflammation. On the contrary, our results showed that ALT and TGF-β1 levels were significantly reduced after vitamin D supplementation. These changes might represent decreases in hepatic fibrogenesis and inflammation.

With regard to HCV viral inhibition, although *in vitro* evidence suggests that 25-hydroxy VD3, but not the active form 1, 25-dihydroxy VD3, inhibits HCV viral replication^37^, we did not observe changes in HCV viral loads in our patients during the 6-week period. However, these findings do not imply that there is no minimal local inhibitory effect of VD in hepatocytes.

The results of this study are not yet clinically beneficial. However, we intended to show that short-term vitamin D replacement, with restoration of vitamin D to physiological levels, changes the levels of specific cytokines and enzymes to favor a fibrolytic state. We chose a 6-week treatment duration because most patients’ VD levels have been shown to be restored after initial doses. We postulated that 6 weeks would be an optimal time to observe immunological changes, which are highly dynamic, as inflammatory processes and viral replication occur in spans of hours and days, respectively. Immunological imbalances caused by viral hepatitis, chronic hepatitis or hepatic fibrogenesis are dynamic processes. However, hepatic scars in liver tissues could take years to form. It will be of interest to determine whether VD supplementation has protective effects on or reverses hepatic scars during or after specific therapies in a future long-term study.

Our study has some limitations; first, it was based on serum markers that do not always represent hepatic fibrogenic events. In addition, several factors are known to influence the VD status of patients. Therefore, the trial was conducted in a double-blinded, placebo-controlled fashion to reduce most bias. To demonstrate the benefit of VD in this situation, the comparison of paired biopsies between groups would be ideal; however, a biopsy both before and after CHC treatment would be ethically controversial. Therefore, this study is considered a bridging study from experimental and animal models to humans and was performed to evaluate the benefits of VD in a fibrotic liver condition using serum markers as surrogate markers for fibrosis. Further studies should focus on the effect of VD or VD derivatives in cryptogenic or non-alcoholic fatty liver disease, as these diseases still have no curative options.

This study used CHC patients as a model to support previous findings *in vitro* and in animal models showing that VD has an important role in the reversal of hepatic fibrogenesis.

## Methods

The CONSORT checklist and a detailed protocol of this trial are available as related manuscript file (Related manuscript).

### Patients and study design

**Figure 4:**
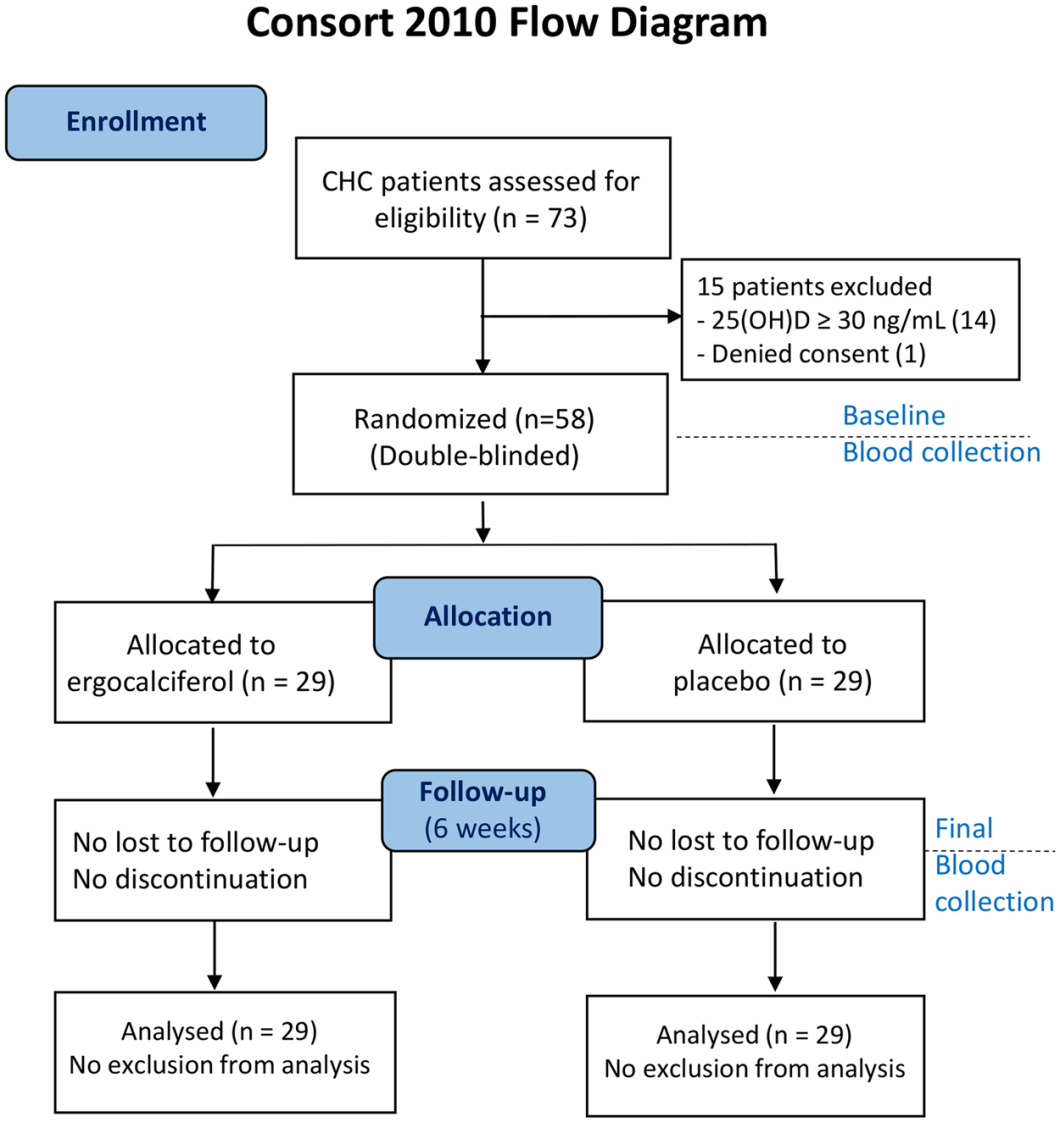
CONSORT 2010 flow diagram^39^, CHC patients who were screened, enrolled and monitored. Of 65 patients who were screened for VD deficiency, 7 were excluded, and 58 were randomly assigned to receive placebo or VD supplement; both groups were evaluated after 6 weeks of supplementation.

A randomized, double-blinded, placebo-controlled, interventional study was carried out in CHC patients with VD deficiency. The criteria for “VD deficiency” used in this trial included VD insufficiency (VD levels between 20–29 ng/mL) and VD deficiency (VD levels less than 20 ng/mL) according to the Endocrine Society Practice Guidelines^38^. The diagnosis of CHC was based on the detection of hepatitis C virus (HCV) RNA. The inclusion criteria consisted of patient age between 18 to 70 years old, naïve cases or non-responder cases of CHC without decompensated cirrhosis, and serum 25(OH)VD levels less than 30 ng/mL. Patients with decompensated cirrhosis, human immunodeficiency virus infection, autoimmune diseases, active infections from other pathogens, a history of steroid or immunosuppressive therapy, or a history of interferon treatment within 12 months were excluded from the study. Sixty-five CHC patients were screened for VD levels, and 58 patients who had VD deficiency without any exclusion criteria were enrolled and randomized into the VD (29 cases) or placebo (29 cases) group (Fig. 4).

### Randomization

The enrolled patients were randomized into two groups with a 1:1 allocation using a random block size of 4, which was generated by computer software based on www.randomisation.com. The randomization was performed by a research assistant who was not involved in the clinical trial. Upon enrollment, the patients were stratified into two groups (A and B) with the details of the drugs placed in sealed envelopes.

### Sample size calculation

The sample size calculation was based on a previous study that evaluated mean changes in serum TGF-β1 in patients with CHC who did or did not respond to interferon therapy^40^. The estimated sample size for comparing two mean values of independent data sets was 24 cases in each group based on 95% power (β = 0.05) and considering a *p*-value of < 0.05 to be statistically significant. With expected ten percent dropout rate, at least 27 cases are estimated in each arm. The formula for sample size calculation is submitted in related manuscript file (Related manuscript: protocol). Sample size calculations were performed using Power and Sample Size Calculations, PS version 3.0.4.3^41^.

### Intervention

Vitamin D2 (VD2, ergocalciferol) and a placebo were prepared in identical capsules of the same weight by the Department of Pharmacy at King Chulalongkorn Memorial Hospital. All investigators and patients were blinded to the type of medication used until the end of the study. Each capsule of VD2 contained 20,000 international units (IU) ergocalciferol.

This VD2 dose has been previously demonstrated to effectively increase VD levels in CHC patients within a 6-week period (preliminary data obtained before the clinical trial was conducted). Patients with VD levels ranging from 20 to less than 30 ng/mL were administered VD2 or equivalent placebo capsules at a dosage of 60,000 IU per week in two divided doses. Two capsules (2 × 20,000 IU) were given to these patients on Monday, and 1 capsule was given to them (20,000 IU) on Friday. Patients with VD levels ranging from 10 to less than 20 ng/mL were administered VD2 or equivalent placebo capsules at a dosage of 80,000 IU per week in two divided doses. Two capsules (2 × 20,000 IU) were given to these patients on both Monday and Friday. Patients with VD levels below 10 ng/mL were administered VD2 or equivalent placebo capsules at a dosage of 100,000 IU per week in two divided doses. Three capsules (3 × 20,000 IU) were given to these patients on Monday, and 2 capsules were given to them (2 × 20,000 IU) on Friday (Related manuscript: protocol).

The patients in this study were enrolled and followed up for 6 weeks during the interventional period, which lasted for three seasons in Thailand. Notably, there was not much seasonal variation in sun exposure that might have caused changes in the patients’ vitamin D levels. In this regard, all patients enrolled in this study were explained in detail regarding the study protocol and asked not to change their usual daily activities or purposefully try to increase their activities that would increase in sun exposure. However, this type of bias could be diminished by VD changes in the placebo group.

Baseline clinical characteristics of the patients and fibrosis-4 (FIB4) index values were recorded. Samples of whole blood were collected at baseline and at the end of the 6-week follow-up period and kept at −70 °C until analysis. Serum samples were used for the analysis of 25(OH)VD, TGF-β1, TIMP-1, MMP2 and MMP9 levels.

Pill counts and patient interviews were used to assess adherence to the prescribed medications. At the end of the trial, patients who remained in a VD insufficient state received VD supplementation as a standard of care.

### Laboratory analysis

#### Vitamin D assays

The VD level was determined in serum samples using the Liaison 25 OH vitamin D total assay (DiaSorin, Saluggia, Italy), which was performed on the LIAISON® chemiluminescence analyzer according to the manufacturer’s instructions. The final concentration is reported in ng/mL.

### Measurement of TGF-β_1_ levels

The serum concentrations of human activated TGF-β1 protein were measured with a quantitative sandwich enzyme-linked immunosorbent assay (ELISA) technique according to the manufacturer’s instructions (Quantikine® ELISA, R&D Systems, Minneapolis, MN, USA). Before the assay, the latent TGF-β1 contained in patients’ serum was activated to the immunoreactive form using acid activation and neutralization. The results were calculated by reference to the standard curve.

### Measurement of TIMP-1 levels

The serum concentrations of human TIMP-1 were measured via the quantitative sandwich ELISA technique according to the manufacturer’s instructions (Quantikine® ELISA). The results were calculated by reference to the standard curve.

### Measurement of MMP2 and MMP9 levels

The serum concentrations of human MMP2 and MMP9 protein were measured via the Luminex bead-based multiplex assay with an MMP panel according to the manufacturer’s instructions (Luminex® Performance assay, R&D Systems). The kits were run on a Luminex® 100™ Bioanalyzer (Luminex Corp., Austin, TX, USA) according to the kit manufacturer’s instructions.

### HCV RNA quantification

To evaluate the changes in HCV viral loads after VD or placebo supplementation in each group, we used plasma samples that had been stored at −80 °C and had not been previously thawed. HCV RNA viral loads were determined using Abbott RealTime HCV assays according to the manufacturer’s instructions, and they are expressed as international units per milliliter (IU/mL). The range of viral detection was between 12 and 1 × 10^8^ IU/mL.

### Study endpoint

The primary outcome was the effect of VD supplementation on changes in TGF-β1, TIMP-1, MMP2 and MMP9 levels relative to the placebo group at the 6-week follow-up.

### Statistical analysis

Baseline characteristics, including clinical and laboratory data of the patients, were presented as the percentage or mean ± SD. Continuous variables with a normal distribution were compared within groups (pre- and post-treatment) with a dependent *t*-test; variables with a skewed distribution were compared with a Wilcoxon signed-rank test. Continuous variables with a normal distribution were compared between two groups with an independent *t*-test; variables with a skewed distribution were compared with the Mann-Whitney *U*-test. Categorical variables were compared between groups was performed through ANCOVA. In ANCOVA analysis, the dependent variable is the post-test measure, and the pre-test measure was a covariate and controlled for. And also controlled were cirrhosis, gender and genotype status. Cohen’s d effect size power analysis was used to measure the strength of VD supplementation on the changes in serum markers between groups. The magnitudes of effects were stratified as 0.2 small, 0.5 medium and 0.8 large^42^. All statistical data were analyzed with SPSS version 17.0 software, SPSS Inc., Chicago, IL, USA and SAS version 9.4, SAS Institute Inc., USA. All data set of this study is deposited in supplementary file (Supplementary information).

### Ethics approval and consent to participate

The study was reviewed and approved by the Ethics Committee, Institutional Review Board (IRB) at Chulalongkorn University, Bangkok, Thailand in accordance with the Declaration of Helsinki (1989) of the World Medical Association (IRB No: 044/57 and 201/57). The clinical trial was retrospectively registered with the Thai Clinical Trials Registry (TCTR) based on World Health Organization criteria on 2 November 2016 (TCTR20161103003). The trial was conducted between February 2014 and January 2015. All patients enrolled in this study provided written informed consent and had consent for publication.

### Availability of data and materials

The datasets analyzed and additional supplementary tables in the current study are available in Supplementary information. The consort 2010 checklist and research protocol are submitted in related manuscript file.

## Electronic supplementary material

Supplementary information
