# Supplementary material for: Vitamin D supplementation improves serum markers associated with hepatic fibrogenesis in chronic hepatitis C patients: A randomized, double-blind, placebo-controlled study
Source: Sci Rep. 2017 Aug 21;7:8905. doi: 10.1038/s41598-017-09512-7 (PMC5566364; doi:10.1038/s41598-017-09512-7)
Supplement: Supplementary file 1 — Supplementary information [file 41598_2017_9512_MOESM1_ESM.pdf]

Supplementary information

Vitamin D supplementation improves serum markers associated with hepatic fibrogenesis in chronic hepatitis C patients: A randomized, double-blind, placebo-controlled study

Piyawat Komolmit* [1, 2,], Sayamon Kimtrakool[3], Sirinporn Suksawatamnuay[1, 2], Kessarin Thanapirom[1, 2], Kanita Chattrasophon[4], Panarat Thaimai[1], Chintana Chirathaworn[5], and Yong Poovorawan[6]

1 Division of Gastroenterology, Department of Medicine, Faculty of Medicine, Chulalongkorn University, Bangkok, Thailand
2 Center of Excellence in Liver Diseases: King Chulalongkorn Memorial Hospital, Thai Red Cross Society, Bangkok, Thailand
3 Gastroenterology Unit, Department of Medicine, Lerdsin Hospital, Bangkok, Thailand
4 Gastroenterology Unit, Department of Medicine, Taksin Hospital, Bangkok, Thailand
5 Division of Immunology, Department of Microbiology, Faculty of Medicine, Chulalongkorn University, Bangkok, Thailand
6 Center of Excellence in Clinical Virology, Faculty of Medicine, Chulalongkorn University, Bangkok, Thailand

* Corresponding author: Piyawat Komolmit, M.D., Ph.D.
Division of Gastroenterology, Department of Medicine, Faculty of Medicine, Chulalongkorn University, Bangkok 10330, Thailand
Tel: +662-2564265, +669-47825195, email: pkomolmit@yahoo.co.uk

Table of contents **page**

1. Supplementary tables 2

2. Data set 9

1. SUPPLEMENTARY TABLES

Supplementary Table S1. VD levels at baseline (A), 6-week follow up (B) and the delta changes of VD at 6 weeks (C); VD levels are in ng/mL

(A) VD levels at baseline

Groups	VD levels	N	Mean ± SD	Min	Max	Range
VD Replacement	< 20	11	14.8 ± 4.4	6.6	19.7	13.1
	20 to < 30	18	23.1 ± 2.5	20.0	29.6	9.6
	Total	29	19.9 ± 5.3	6.6	29.6	23.0
Placebo	< 20	15	14.6 ± 3.3	9.5	19.5	10
	20 to < 30	14	23.8 ± 2.2	20.1	27.0	6.9
	Total	29	19.0 ± 5.5	9.5	27.0	17.5

(B) VD levels at 6-week follow up

Groups	VD levels	N	Mean ± SD	Min	Max	Range
VD Replacement	< 20	11	45.4 ± 16.1	24.0	70.8	46.8
	20 to < 30	18	45.8 ± 10.5	27.9	64.4	36.5
	Total	29	45.6 ± 12.6	24.0	70.8	46.8
Placebo	< 20	15	15.6 ± 3.9	9.8	22.7	12.9
	20 to < 30	14	23.9 ± 2.5	20.1	27.9	8.8
	Total	29	19.6 ± 5.3	9.5	27.9	18.1

(C) The delta changes of VD at 6 weeks

Groups	VD levels	N	Mean ± SD	Min	Max	Range
VD Replacement	< 20	11	30.6 ± 16.1	8.4	60.2	51.8
	20 to < 30	18	22.7 ± 11.2	-1.7	40.1	41.8
	Total	29	25.7 ± 13.5	-1.7	60.2	61.9
Placebo	< 20	15	1.0 ± 2.8	-3.5	6.6	10.1
	20 to < 30	14	0.07 ± 2.6	-5.4	3.9	9.3
	Total	29	0.6 ± 2.7	-5.4	6.6	12.0

Supplementary Table S2. VD levels at baseline, and the delta changes of VD and serum markers

The data are showed in mean ± SD. The levels of VD and other markers are shown in ng/mL, except for the levels of TGF ß1 are shown in pg/mL.

Groups	VD levels at baseline	Changes in the levels at 6 weeks (delta changes)				
		VD	TGF ß1	TIMP-1	MMP2	MMP9
VD Replacement	< 20	30.6 ± 16.1	-23.1 ± 136.0	-0.2 ± 0.6	57.8 ± 152.0	20.2 ± 56.4
	20 to < 30	22.7 ± 11.2	-25.5 ± 101.0	-0.1 ± 0.3	48.8 ± 110.3	41.2 ± 25.3
	Total	25.7 ± 13.5	-24 ± 113.2	-0.1 ± 0.4	51.7 ± 121.4	34.2 ± 38.6
Placebo	< 20	1.0 ± 2.8	16.4 ± 79.2	0.4 ± 1.1	-45.8 ± 144.6	-24.9 ± 22.6
	20 to < 30	0.07 ± 2.6	100.8 ± 119.0	0.3 ± 0.3	-36.5 ± 109.6	-21.5 ± 32.0
	Total	0.6 ± 2.7	57.1 ± 107.5	0.4 ± 0.8	-41.9 ± 128.6	-23.4 ± 26.6

Supplementary Table S3. The characteristic of 3 patients in VD group whose vitamin D levels remained lower than 30 ng/mL after 6-week replacement. (A) the basic characteristics, (B) the changes of the markers after 6-week VD replacement

(A)

Case	Age	Gender	Genotype	Viral load (IU/mL)	Cirrhosis	Pre VD Level (ng/mL)	Post VD level (ng/mL)	VD changes (ng/mL)
1	53	M	3	3244589	yes	15.8	25.9	10.1
2*	47	M	3	2175432	no	29.6	27.9	-1.7
3	54	F	3	1713	yes	15.6	24.0	8.4

(B)

Case	TGF ß1 changes**	TIMP1 changes**	MMP2 changes*	MMP9 changes**
1	- 371	- 1.01	91.49	- 106
2	- 126	- 0.3	21.26	65.6
3	- 17.3	0.38	NA	62.7

* case number 2 had a reduction of VD level of 1.7 ng/mL after 6 weeks replacement

** The changes in favor of decreasing fibrogenesis: reduction (minus) of TGF b1 and TIMP-1 and increment (plus) of in MMP2 and MMP9 levels

Supplementary Table S4. Changes of transaminase levels and hepatitis C viral load after 6-week vitamin D supplementation in vitamin D supplement group (A), placebo group (B)

(A)

Parameters (n*)	VD supplement group				
	Baseline	At 6 weeks	Mean changes	p-value	95%CI
AST** (22)	90.9 ± 65.6	83.7 ± 68.9	-7.2 ± 67.4	0.62	- 37.1 – 22.6
ALT** (22)	116.7 ± 96.6	78.0 ± 60.3	-38.1 ± 70.5	0.02	-69.3 – (-6.8)

(B)

Parameters (n*)	Placebo group				
	Baseline	At 6 weeks	Mean changes	p-value	95%CI
AST** (23)	83.4± 50.6	82.1 ± 60.8	-1.32 ± 49.4	0.90	-23.2 – 20.6
ALT** (23)	102.5 ± 69.9	101.8 ± 74.9	-0.73 ± 34.7	0.92	-16.1 – 14.7

* Number of the patients who had paired pre-and post-supplement levels in each group were used for calculation.

** IU/mL

Supplementary Table S5. Changes of hepatitis C viral loads

(A) comparison of HCV viral load between at the results from the beginning of the trial (original tests) and the stored plasma.

Mean HCV RNA viral load at baseline (log IU/mL)				
Original tests (51 cases)	Stored plasma (51 cases)	Mean changes	p-value	95%CI
5.57 ± 0.83	5.29 ± 1.00	-0.28 ± 0.90	0.028	-0.54, -0.03

(B) Hepatitis C viral loads at baseline and after 6-week of vitamin D or placebo supplementation (assessed in stored plasma)

Groups (n)	Mean HCV viral load (log IU/mL) at				
	Baseline	At 6 weeks	Mean changes	p-value	95%CI
VD (27)	5.29 ± 0.94	5.3 ± 0.88	0.01 ± 0.58	0.93	-0.22 – 0.24
placebo (27)	5.26 ± 1.07	5.3 ± 1.15	0.04 ± 1.00	0.82	-0.35 – 0.44

Supplementary Table S6. Changes in the mean levels of VD, TGF ß1, TIMP-1, MMP2 and MMP9 after 6-weeks VD supplementation of different variables including genders, vitamin D levels at baseline, hepatitis C genotypes and cirrhotic status (only in vitamin D supplement group)

variables	Subgroups (n) and p values	Changes in mean levels (delta changes)				
		VD	TGF β1	TIMP-1	MMP2	MMP9
Gender	M (21)	26.9 ± 10.6	-31.6 ± 77.9	-0.20 ± 0.36	41.4 ± 109.1	31.8 ± 29.2
	F (8)	22.6 ± 17.4	-6.3 ± 152.4	0.07 ± 0.43	79.2 ± 64.6	40.2 ± 51.7
	p values	0.21	0.14	0.74	0.53	0.66
VD levels at baseline	< 20 (11)	30.6 ± 16.1	-23.1 ± 136.0	-0.17 ± 0.57	57.8 ± 152.0	20.2 ± 56.4
	20 to < 30 (18)	22.6 ± 11.2	-25.5 ±101.0	-0.10 ± 0.30	48.8 ±110.3	41.2 ± 25.3
	p values	0.34	0.50	0.08	0.37	0.06
Genotypes**	1 (15)	26.9 ± 10.6	-34.9 ± 77.9	-0.05 ± 0.36	48.9 ± 109.1	38.5 ± 29.2
	3 (12)	23.9 ±17.4	-26.9 ± 152.4	-0.30 ± 0.43	-5.9 ± 64.6	27.7 ± 51.7
	p values	0.09	0.08	0.24	0.22	0.22
Cirrhosis	Yes (11)	19.2 ± 29.7	-23.6 ± 147.9	-0.21 ± 0.45	12.8 ± 82.6	28.8 ± 54.3
	No (18)	29.7 ± 14.7	-25.2 ± 90.6	-0.08 ± 0.41	81.9 ± 134.4	37.5 ± 27.0
	p values	0.14	0.32	0.38	0.28	0.25

* Levels are in ng/mL, except for TGF β1 levels are in pg/mL

** Two cases of genotype 2 and 6 were not included for analysis.

2. DATA SET

Person	HN	Age	Sex_forma	Sex	Genotype	G1	G1_3	Pre_HCV_\	Log_Pre_H
1	7646956	39	F	2	3	2	2	2710496	6.43
2	9956856	38	M	1	1	1	1	59083	4.77
3	5774055	43	M	1	3	2	2	714135	5.85
4	10120356	53	M	1	3	2	2	1333896	6.13
5	477257	47	M	1	3	2	2	16236	4.21
6	519157	58	F	2	3	2	2	546924	5.74
7	6990956	37	M	1	1	1	1	1669871	6.22
8	9528054	45	F	2	1	1	1	421815	5.63
9	1801857	63	F	2	1	1	1	6383	3.81
10	1826857	55	M	1	3	2	2	5547	3.74
11	6887429	59	F	2	1	1	1	485398	5.69
12	616457	51	M	1	1	1	1	611938	5.79
13	1238557	55	M	1	3	2	2	1461	3.16
14	30413657	39	M	1	3	2	2	38774	4.59
15	4559554	26	M	1	1	1	1	906649	5.96
16	980547	56	M	1	1	1	1	176634	5.25
17	6396142	53	M	1	3	2	2	348983	5.54
18	13220439	62	M	1	1	1	1	1391280	6.14
19	10491256	46	F	2	3	2	2	407266	5.61
20	4384043	70	F	2	1	1	1	4188641	6.62
21	30612157	35	M	1	1	1	1	2090470	6.32
22	3350349	65	F	2	1	1	1	1855302	6.27
23	5884356	39	F	2	6	2	3	85713	4.93
24	11707844	62	F	2	1	1	1	3441179	6.54
25	1313855	56	F	2	1	1	1	2544543	6.41
26	30910757	28	M	1	1	1	1	624962	5.80
27	#NULL!	45	M	1	3	2	2	1371883	6.14
28	#NULL!	52	M	1	1	1	1	1234768	6.09
29	#NULL!	41	M	1	2	2	3	14410	4.16
30	#NULL!	58	M	1	3	2	2	1043313	6.02
31	30806757	60	F	2	1	1	1	1043313	6.02
32	5633456	56	M	1	1	1	1	670411	5.83
33	7002252	48	M	1	3	2	2	12090	4.08
34	8282555	69	M	1	3	2	2	40727	4.61
35	6753653	43	M	1	3	2	2	180393	5.26
36	2162555	56	M	1	3	2	2	#NULL!	#NULL!
37	8455354	55	M	1	3	2	2	4992	3.70
38	8965956	28	F	2	3	2	2	235545	5.37
39	633256	68	F	2	1	1	1	8047	3.91
40	11221152	45	M	1	3	2	2	73965	4.87
41	31470257	48	M	1	1	1	1	55856	4.75
42	11875940	27	F	2	1	1	1	651848	5.81
43	#NULL!	43	M	1	1	1	1	755389	5.88
44	31036957	50	F	2	1	1	1	68468	4.84
45	31983957	57	M	1	3	2	2	129696	5.11
46	1910751	62	F	2	1	1	1	1050663	6.02

47	10909555	53	M	1	3	2	2	1949	3.29
48	6630650	52	M	1	1	1	1	3639968	6.56
49	33320357	61	M	1	3	2	2	286707	5.46
50	5442255	50	F	2	3	2	2	670411	5.83
51	1631857	57	M	1	1	1	1	499	2.70
52	5784354	54	F	2	3	2	2	7934	3.90
53	10204249	57	M	1	1	1	1	#NULL!	#NULL!
54	30443757	39	F	2	1	1	1	7449	3.87
55	#NULL!	50	M	1	3	2	2	#NULL!	#NULL!
56	2854557	59	F	2	1	1	1	2105197	6.32
57	33757357	42	F	2	1	1	1	320789	5.51
58	33825057	55	M	1	1	1	1	#NULL!	#NULL!

Post_HCV_	Log_Post_l	HCV_VL	Log7	Fibroscan	Significant_	Underlying	Status_forı	Naive	Cirrhosis_f
1681635	6.23	2985070	1	7.60	1	N	New	1	N
110358	5.04	261019	2	16.60	1	N	New	1	N
750105	5.88	2082352	1	8.50	1	N	New	1	N
1261048	6.10	3244589	1	48.80	1	HT	New	1	Y
105066	5.02	2175432	1	18.40	1	HT	New	1	N
719166	5.86	146548	2	4.40	2	N	New	1	N
1088196	6.04	589257	2	6.80	2	N	New	1	N
377000	5.58	112151	2	15.10	1	DLP	New	1	Y
1287888	6.11	810747	2	14.50	1	N	New	1	N
6751	3.83	5512	2	26.30	1	N	New	1	Y
611938	5.79	280055	2	16.30	1	N	New	1	Y
62059	4.79	464905	2	4.30	2	N	New	1	N
8160	3.91	22689	2	17.20	1	HT	New	1	Y
13061	4.12	147583	2	8.20	1	N	New	1	N
1693482	6.23	354167	2	6.80	2	N	New	1	N
3465422	6.54	330866	2	8.00	1	N	Fail	2	N
126105	5.10	199755	2	9.10	1	N	Fail	2	Y
88775	4.95	2779077	1	19.40	1	N	New	1	N
488817	5.69	259188	2	9.00	1	N	New	1	N
2526742	6.40	2905447	1	11.20	1	HT	Fail	2	N
5027377	6.70	4790000	1	10.10	1	N	New	1	N
900306	5.95	651000	2	14.20	1	N	Fail	2	Y
193512	5.29	338000	2	13.00	1	N	New	1	N
2090470	6.32	700000	2	6.80	2	N	New	1	N
1461356	6.16	1844394	1	21.50	1	N	New	1	Y
233897	5.37	#NULL!	#NULL!	8.70	1	N	New	1	N
709140	5.85	5394957	1	8.30	1	N	New	1	N
679891	5.83	1563728	1	30.80	1	HT	New	1	Y
656	2.82	668604	2	9.20	1	N	New	1	N
1143007	6.06	1645146	1	21.80	1	N	New	1	N
34654	4.54	869877	2	16.00	1	N	New	1	N
1080584	6.03	4799992	1	21.30	1	HT	New	1	Y
13815	4.14	1322132	1	15.30	1	N	Fail	2	N
9726	3.99	81389	2	17.30	1	N	Fail	2	N
301148	5.48	211391	2	11.30	1	N	New	1	N
#NULL!	#NULL!	2082352	1	#NULL!	2	N	New	1	Y
14	1.15	#NULL!	#NULL!	3.90	2	N	New	1	N
55078	4.74	144069	2	6.40	2	N	New	1	N
22425	4.35	181907	2	15.00	1	N	New	1	Y
232261	5.37	289916	2	20.80	1	N	New	1	Y
188153	5.27	459404	2	23.10	1	N	New	1	Y
356411	5.55	1703623	1	21.30	1	N	New	1	N
675134	5.83	4447651	1	7.00	1	N	New	1	N
21052	4.32	159807	2	29.50	1	N	New	1	Y
65644	4.82	543708	2	30.30	1	N	New	1	Y
782374	5.89	538000	2	6.40	2	N	New	1	N

4923	3.69	244977	2	27.30	1	N	Fail	2	Y
1829435	6.26	1812	2	21.60	1	N	Fail	2	Y
611938	5.79	379262	2	11.00	1	N	New	1	N
1175558	6.07	264252	2	31.20	1	N	New	1	Y
90031	4.95	3251	2	24.70	1	N	New	1	Y
374363	5.57	1713	2	39.70	1	N	New	1	Y
#NULL!	#NULL!	#NULL!	#NULL!	6.10	2	N	New	1	N
233897	5.37	62553	2	7.80	1	N	New	1	N
#NULL!	#NULL!	#NULL!	#NULL!	21.50	1	N	New	1	N
993283	6.00	1232944	1	17.30	1	N	New	1	N
546924	5.74	#NULL!	#NULL!	8.20	1	N	New	1	N
#NULL!	#NULL!	257831	2	7.40	1	N	New	1	N

Cirrhosis	Interventic	Interventic	Hb	Wbc	Platelet	PT	INR	TB	DB
2	BB	2	13.1	6870	294000	12.3	1.08	0.66	0.35
2	BB	2	15.5	6670	215000	14.0	1.24	0.85	0.41
2	AA	1	13.9	6490	234000	13.0	1.10	0.76	0.28
1	AA	1	14.6	4320	72000	14.0	1.20	0.90	0.78
2	AA	1	15.0	6710	152000	13.2	1.10	0.97	0.26
2	BB	2	14.2	7830	186000	12.0	0.90	1.65	0.45
2	AA	1	14.8	7320	222000	12.9	1.10	1.10	0.40
1	BB	2	13.4	5110	126000	14.3	1.20	0.70	0.48
2	BB	2	13.0	5920	107000	13.0	1.17	1.00	0.40
1	AA	1	14.9	7380	87000	14.9	1.28	1.17	0.43
1	BB	2	11.9	4330	128000	13.0	1.00	1.00	0.30
2	AA	1	17.0	10050	246000	13.7	1.19	1.70	0.50
1	AA	1	14.0	4300	106000	14.9	1.30	0.70	0.40
2	BB	2	16.9	6880	221000	12.0	1.00	0.97	0.38
2	AA	1	15.9	8110	302000	12.3	1.00	1.50	0.40
2	BB	2	15.1	3650	126000	13.3	1.10	0.70	0.30
1	AA	1	14.0	4610	153000	13.9	1.20	0.95	0.36
2	BB	2	14.0	5230	180000	12.8	1.00	0.56	0.21
2	BB	2	13.4	5600	321000	13.4	1.17	1.05	0.52
2	AA	1	13.2	5750	252000	12.2	1.08	1.06	0.27
2	BB	2	16.0	6890	184000	13.8	1.20	1.14	0.50
1	BB	2	13.6	6840	162000	13.7	1.19	0.55	0.21
2	AA	1	11.4	6500	256000	12.9	1.05	0.50	0.10
2	AA	1	13.2	6590	238000	12.4	1.09	1.08	0.42
1	BB	2	12.9	6990	142000	13.2	1.16	1.23	0.45
2	AA	1	14.4	10950	174000	13.3	1.10	1.08	0.48
2	BB	2	13.0	7800	233000	12.0	0.90	0.90	0.30
1	AA	1	14.0	9600	142000	13.2	1.10	1.20	0.50
2	AA	1	14.9	6000	230000	12.1	0.90	0.60	0.20
2	BB	2	15.0	7000	200000	12.2	0.90	1.00	0.40
2	BB	2	12.4	5380	118000	14.0	1.22	1.40	0.60
1	AA	1	11.8	4280	74000	17.0	1.50	1.50	0.80
2	BB	2	16.0	7700	131000	13.0	1.10	0.76	0.33
2	AA	1	15.2	7310	274000	11.0	0.80	0.98	0.37
2	AA	1	15.8	7920	217000	13.4	1.17	1.70	0.80
1	AA	1	13.6	4750	206000	12.0	0.80	1.06	0.33
2	BB	2	13.2	10840	321000	12.0	0.90	0.65	0.24
2	BB	2	13.9	6300	276000	11.4	1.01	0.46	0.20
1	AA	1	13.0	3090	133000	16.0	1.30	2.00	1.00
1	BB	2	14.5	4690	126000	13.5	1.19	1.10	0.40
1	AA	1	14.3	8760	107000	13.8	1.20	0.70	0.30
2	AA	1	13.0	6860	162000	1.3	1.40	1.40	0.75
2	AA	1	14.0	6700	346000	12.0	1.00	0.90	0.30
1	BB	2	11.0	3720	30000	14.8	1.28	2.10	0.90
1	AA	1	11.2	6110	91000	16.0	1.30	1.75	1.00
2	BB	2	13.7	8470	451000	11.3	1.00	1.30	0.60

1	BB	2	13.2	3160	63000	14.0	1.20	1.50	0.60
1	BB	2	14.0	5660	79000	14.0	1.21	2.50	1.00
2	AA	1	13.6	10100	219000	12.0	1.00	1.11	0.50
1	BB	2	13.9	2530	32000	19.0	1.60	2.00	1.70
1	BB	2	13.6	5170	78000	12.5	1.00	1.80	1.00
1	AA	1	12.7	2930	74000	15.0	1.30	1.80	0.80
2	AA	1	12.9	8410	246000	11.0	0.90	0.65	0.26
2	BB	2	13.5	8800	307000	14.0	1.20	0.60	0.30
2	BB	2	12.0	6270	120000	11.0	1.00	0.50	0.20
2	AA	1	13.5	11570	335000	11.0	0.90	0.70	0.50
2	AA	1	14.0	5150	189000	13.0	1.10	0.80	0.36
2	BB	2	13.0	6660	161000	12.4	1.09	1.28	0.40

Albumin	Globulin	PreSGOT	PreSGPT	PreALP	PostSGOT	PostSGPT	PostALP	Cr	Pre_VitD
4.3	3.7	45.00	47.00	50.00	37	41	55	0.90	14.10
4.4	4.1	68.00	103.00	80.00	73	106	#NULL!	0.90	24.50
4.5	3.9	40.00	59.00	54.00	#NULL!	#NULL!	#NULL!	0.80	22.00
3.7	3.7	433.00	400.00	70.00	#NULL!	#NULL!	#NULL!	1.00	15.80
4.3	3.4	269.00	392.00	173.00	178	232	#NULL!	0.90	29.60
3.9	4.1	20.00	23.00	20.00	#NULL!	#NULL!	#NULL!	1.40	21.60
4.3	3.9	34.00	72.00	94.00	24	44	82	1.00	22.10
3.7	3.9	116.00	116.00	114.00	90	97	#NULL!	0.60	16.10
3.9	3.4	33.00	28.00	64.00	37	36	57	0.60	9.50
3.8	4.8	65.00	60.00	101.00	47	40	95	0.80	19.60
3.9	3.2	40.00	45.00	89.00	50	62	#NULL!	0.80	10.50
4.1	3.5	51.00	68.00	79.00	41	47	68	0.90	17.90
2.4	4.9	86.00	60.00	208.00	65	39	184	0.80	27.50
3.9	3.0	101.00	173.00	101.00	66	120	70	0.80	23.90
4.8	3.3	42.00	89.00	83.00	24	29	#NULL!	1.20	22.70
3.9	3.0	94.00	54.00	99.00	43	78	#NULL!	0.90	25.90
3.7	3.6	264.00	380.00	113.00	114	149	107	1.20	21.30
3.8	4.4	55.00	83.00	67.00	55	83	67	1.10	21.20
3.5	5.6	34.00	19.00	131.00	#NULL!	#NULL!	#NULL!	0.60	10.10
4.0	3.6	61.00	51.00	68.00	43	37	65	0.70	16.00
4.1	4.2	68.00	133.00	56.00	48	73	46	1.10	18.90
3.8	2.2	21.00	17.00	74.00	25	26	78	0.68	15.60
3.0	3.4	51.00	55.00	87.00	#NULL!	#NULL!	#NULL!	0.70	19.00
4.0	4.0	102.00	130.00	97.00	114	113	102	0.78	20.20
3.9	4.0	90.00	104.00	63.00	93	104	#NULL!	0.70	26.30
4.2	3.8	74.00	204.00	69.00	23	33	56	1.10	22.20
4.7	3.5	53.00	71.00	93.00	#NULL!	#NULL!	#NULL!	0.90	19.50
4.2	3.8	192.00	188.00	80.00	#NULL!	#NULL!	#NULL!	0.80	19.70
4.1	3.9	25.00	80.00	64.00	#NULL!	#NULL!	#NULL!	1.00	23.00
4.3	4.0	246.00	241.00	110.00	#NULL!	#NULL!	#NULL!	0.90	24.00
3.6	3.7	203.00	255.00	150.00	236	274	144	0.70	13.90
3.5	4.4	96.00	103.00	129.00	76	69	76	1.00	20.00
3.8	3.7	107.00	80.00	124.00	86	51	106	0.70	23.00
4.4	3.8	91.00	121.00	101.00	#NULL!	56	113	0.90	24.30
4.1	3.9	47.00	93.00	66.00	41	78	61	1.00	23.40
3.9	4.7	49.00	122.00	66.00	55	43	71	1.00	20.20
3.8	2.9	16.00	13.00	81.00	21	14	#NULL!	0.70	20.10
4.4	2.8	17.00	21.00	57.00	15	16	54	0.70	19.00
3.0	4.3	105.00	82.00	82.00	114	48	75	0.60	10.10
3.6	4.1	155.00	233.00	137.00	160	240	129	0.80	16.10
3.5	3.9	68.00	66.00	122.00	51	61	138	1.10	21.20
3.5	4.8	181.00	184.00	130.00	154	173	115	0.70	12.20
4.0	3.9	476.00	450.00	100.00	#NULL!	#NULL!	#NULL!	1.00	9.88
3.7	3.8	126.00	101.00	205.00	#NULL!	#NULL!	#NULL!	0.80	16.20
3.0	4.4	94.00	90.00	117.00	128	58	136	0.80	22.70
4.3	4.0	52.00	40.00	107.00	#NULL!	#NULL!	#NULL!	0.50	15.60

4.1	3.1	113.00	142.00	64.00	109	172	58	1.10	26.20
4.0	4.3	101.00	111.00	107.00	98	126	71	1.20	25.00
4.1	3.6	98.00	66.00	97.00	95	72	102	1.00	6.60
3.6	5.0	68.00	51.00	158.00	71	51	163	0.50	11.40
3.2	4.2	185.00	174.00	326.00	80	82	360	0.70	12.00
3.0	4.6	78.00	108.00	131.00	320	217	138	0.90	15.60
4.4	3.9	29.00	22.00	84.00	23	20	79	0.70	23.10
4.5	4.6	61.00	198.00	82.00	244	260	104	0.60	23.90
4.0	4.0	78.00	75.00	80.00	69	128	78	#NULL!	27.00
4.0	3.9	63.00	73.00	#NULL!	67	75	70	0.80	25.80
4.4	3.1	45.00	54.00	55.00	45	54	55	0.60	24.50
4.2	3.7	59.00	140.00	48.00	#NULL!	#NULL!	#NULL!	1.20	21.00

VD_classifi	Classified_	Post_VitD	Responder	Classify_Vi	VitD_chang	Pre_TGFbe	Post_TGFb	TGFbeta_c	Pre_PDGF
1	1	14.50	#NULL!	1	0.40	694.37	630.83	-63.54	111.71
2	2	19.10	#NULL!	1	-5.40	357.98	644.47	286.49	199.55
2	2	56.00	1	2	34.00	620.85	403.30	-217.55	164.67
1	1	25.90	2	1	10.10	886.15	514.74	-371.41	150.87
2	2	27.90	2	1	-1.70	675.50	549.29	-126.21	202.95
2	2	24.20	#NULL!	1	2.60	669.87	669.60	-0.27	231.39
2	2	61.50	1	2	39.40	572.15	539.72	-32.43	179.77
1	1	22.70	#NULL!	1	6.60	821.57	999.70	178.13	101.76
1	1	14.80	#NULL!	1	5.30	423.30	399.58	-23.72	45.91
1	1	49.70	1	2	30.10	195.97	308.86	112.89	53.52
1	1	10.40	#NULL!	1	-0.10	921.85	807.48	-114.37	32.69
1	1	70.80	1	2	52.90	311.14	395.10	83.96	134.19
2	2	34.10	1	2	6.60	833.72	1001.66	167.94	156.40
2	2	24.30	#NULL!	1	0.40	438.97	664.74	225.77	137.68
2	2	52.80	1	2	30.10	823.59	619.09	-204.50	168.80
2	2	24.80	#NULL!	1	-1.10	1127.10	1058.22	-68.88	194.11
2	2	43.10	1	2	21.80	574.82	560.61	-14.21	85.96
2	2	22.00	#NULL!	1	0.80	363.53	451.53	88.00	196.83
1	1	14.30	#NULL!	1	4.20	558.84	584.15	25.31	155.02
1	1	52.60	1	2	36.60	684.91	711.70	26.79	80.16
1	1	18.30	#NULL!	1	-0.60	862.34	754.60	-107.74	140.46
1	1	18.20	#NULL!	1	2.60	785.54	799.48	13.94	180.45
1	1	55.80	1	2	36.80	840.84	900.24	59.40	103.89
2	2	37.30	1	2	17.10	643.82	588.15	-55.67	125.09
2	2	25.80	#NULL!	1	-0.50	790.51	792.50	1.99	111.70
2	2	34.30	1	2	12.10	562.38	537.95	-24.43	199.55
1	1	19.70	#NULL!	1	0.20	506.51	549.88	43.37	181.14
1	1	51.00	1	2	31.30	843.90	875.77	31.87	54.27
2	2	41.00	1	2	18.00	628.17	700.56	72.39	87.40
2	2	25.00	#NULL!	1	1.00	972.20	894.07	-78.13	79.43
1	1	10.40	#NULL!	1	-3.50	284.41	298.96	14.55	106.74
2	2	38.50	1	2	18.50	529.88	504.27	-25.61	41.29
2	2	20.10	#NULL!	1	-2.90	816.53	1071.66	255.13	168.11
2	2	64.40	1	2	40.10	637.36	700.74	63.38	225.98
2	2	58.90	1	2	35.50	1082.22	1014.87	-67.35	248.90
2	2	35.00	1	2	14.80	859.26	966.54	107.28	166.74
2	2	24.00	#NULL!	1	3.90	867.50	1099.06	231.56	168.11
1	1	20.90	#NULL!	1	1.90	879.92	869.26	-10.66	151.56
1	1	30.00	2	2	20.70	1041.58	901.83	-139.75	63.99
1	1	15.10	#NULL!	1	-1.00	412.65	464.01	51.36	269.71
2	2	42.70	1	2	21.50	492.00	378.22	-113.78	108.60
1	1	31.20	1	2	19.00	546.93	550.07	3.14	104.81
1	1	40.90	1	2	31.02	680.16	598.58	-81.58	160.17
1	1	14.30	#NULL!	1	-1.90	115.03	122.31	7.28	0.06
2	2	49.60	1	2	26.90	747.92	750.54	2.62	47.03
1	1	18.10	#NULL!	1	2.50	909.98	1055.63	145.65	120.60

2	2	25.60	#NULL!	1	-0.60	323.62	379.64	56.02	33.09
2	2	22.00	#NULL!	1	-3.00	597.77	696.24	98.47	59.43
1	1	66.80	1	2	60.20	260.76	298.33	37.57	128.78
1	1	12.51	#NULL!	1	1.11	189.40	239.68	50.28	10.23
1	1	9.80	#NULL!	1	-2.20	250.18	286.16	35.98	43.74
1	1	24.00	2	1	8.40	337.45	320.18	-17.27	42.42
2	2	54.80	1	2	31.70	796.56	732.26	-64.30	130.67
2	2	27.00	#NULL!	1	3.10	930.02	1114.61	184.59	123.12
2	2	27.90	1	1	0.90	481.56	531.34	49.78	61.37
2	2	46.70	2	21	20.90	849.23	910.94	61.71	242.98
2	2	45.60	2	21	21.10	618.03	629.47	11.44	63.31
2	2	22.80	1	2	1.80	322.93	403.30	80.37	158.29

Post_PDGF	PDGF_char	Pre_TIMP	Post_TIMP	TIMP_Char	Pre_MMP2	Post_MMF	MMP2_cha	Pre_MMP9	Post_MMF
105.32	-6.39	1.23	1.28	0.05	354.30	326.54	-27.76	169.49	165.38
146.72	-52.83	2.39	2.40	0.01	452.69	469.58	16.89	172.30	173.50
149.49	-15.18	1.54	1.27	-0.27	339.35	316.73	-22.62	162.60	173.88
63.99	-86.88	2.56	1.55	-1.01	600.08	691.57	91.49	267.87	161.55
110.29	-92.66	1.68	1.38	-0.30	502.50	523.76	21.26	125.70	191.31
93.89	-137.50	2.93	3.74	0.81	297.59	347.61	50.02	203.18	193.03
131.40	-48.37	1.84	1.77	-0.07	274.64	275.18	0.54	403.09	444.07
88.85	-12.91	1.81	1.61	-0.20	501.04	446.84	-54.20	370.48	312.47
43.61	-2.30	1.64	1.61	-0.03	445.15	321.93	-123.22	111.89	81.15
61.76	8.24	1.81	1.01	-0.80	410.18	387.38	-22.80	94.05	124.01
27.10	-5.59	1.69	1.69	0.00	426.35	330.20	-96.15	91.68	70.23
114.54	-19.65	1.40	1.34	-0.06	259.75	248.60	-11.15	403.58	455.69
71.38	-85.02	2.84	2.35	-0.49	613.77	552.22	-61.55	92.68	100.63
191.19	53.51	2.19	2.13	-0.06	355.98	311.28	-44.70	159.78	133.08
188.65	19.85	1.44	1.22	-0.22	275.45	366.09	90.64	135.27	202.73
183.87	-10.24	1.94	2.03	0.09	293.44	309.22	15.78	83.30	88.76
106.03	20.07	3.29	2.84	-0.45	445.78	515.60	69.82	87.68	135.67
159.16	-37.67	2.57	2.79	0.22	396.95	203.94	-193.01	106.54	80.43
155.71	0.69	1.33	1.36	0.03	209.08	276.40	67.32	204.14	206.57
122.29	42.13	1.96	3.04	1.08	503.00	#NULL!	#NULL!	96.07	#NULL!
120.88	-19.58	1.19	1.24	0.05	448.44	374.16	-74.28	#NULL!	112.88
132.09	-48.36	2.30	2.42	0.12	295.07	544.33	249.26	270.64	214.73
135.59	31.70	1.98	1.95	-0.03	250.19	540.45	290.26	183.31	228.00
139.77	14.68	1.93	1.55	-0.38	491.22	561.74	70.52	158.97	189.40
124.39	12.69	2.11	2.49	0.38	516.75	348.42	-168.33	133.72	90.35
199.55	0.00	2.46	2.28	-0.18	322.87	287.88	-34.99	169.59	179.70
170.17	-10.97	2.43	2.95	0.52	410.94	560.59	149.65	202.30	195.28
46.68	-7.59	1.59	1.64	0.05	430.46	#NULL!	#NULL!	73.59	#NULL!
98.90	11.50	2.62	3.23	0.61	288.55	596.33	307.78	125.14	162.90
96.04	16.61	1.47	1.65	0.18	429.78	485.45	55.67	61.36	52.81
80.50	-26.24	1.77	1.67	-0.10	745.16	542.05	-203.11	76.01	56.71
3.79	-37.50	2.65	2.50	-0.15	#NULL!	501.64	#NULL!	#NULL!	150.64
106.74	-61.37	2.87	3.33	0.46	588.69	#NULL!	#NULL!	281.19	#NULL!
192.06	-33.92	2.85	2.27	-0.58	565.07	#NULL!	#NULL!	423.52	#NULL!
223.38	-25.52	1.79	2.07	0.28	401.42	380.97	-20.45	212.86	271.17
155.02	-11.72	1.42	1.52	0.10	474.14	371.37	-102.77	143.72	178.06
179.08	10.97	2.04	2.46	0.42	191.73	252.50	60.77	424.75	327.27
114.53	-37.03	2.46	2.39	-0.07	495.52	433.71	-61.81	#NULL!	582.14
50.48	-13.51	2.59	2.30	-0.29	727.13	845.32	118.19	136.95	184.84
107.45	-162.26	2.26	2.64	0.38	391.47	344.20	-47.27	131.91	70.51
97.85	-10.75	2.49	2.81	0.32	500.40	497.33	-3.07	116.37	185.75
78.75	-26.06	2.54	2.39	-0.15	453.96	260.64	-193.32	205.98	190.24
87.68	-72.49	2.85	2.37	-0.48	389.01	520.95	131.94	257.86	304.00
0.98	0.92	0.16	4.41	4.25	688.25	506.41	-181.84	91.42	91.21
50.31	3.28	2.84	2.89	0.05	#NULL!	#NULL!	#NULL!	87.24	152.22
126.26	5.66	2.13	2.67	0.54	494.28	188.24	-306.04	173.01	148.95

50.96	17.87	2.24	2.85	0.61	537.94	#NULL!	#NULL!	118.63	#NULL!
34.44	-24.99	3.47	3.96	0.49	470.53	516.97	46.44	57.43	72.65
125.63	-3.15	4.10	3.54	-0.56	539.31	#NULL!	#NULL!	202.37	#NULL!
12.39	2.16	1.53	1.77	0.24	879.49	#NULL!	#NULL!	57.84	#NULL!
36.44	-7.30	2.42	2.50	0.08	192.60	260.31	67.71	69.98	50.87
39.11	-3.31	1.96	2.34	0.38	#NULL!	765.62	#NULL!	99.23	161.93
111.76	-18.91	2.30	2.17	-0.13	200.23	428.21	227.98	198.59	200.45
106.71	-16.41	1.57	2.23	0.66	254.15	#NULL!	#NULL!	176.72	#NULL!
65.25	3.88	2.10	2.68	0.58	588.46	383.98	-204.48	123.65	98.79
255.58	12.60	2.57	2.51	-0.06	395.49	547.08	151.59	296.14	324.42
62.02	-1.29	1.66	1.67	0.01	176.38	214.43	38.05	75.01	157.84
88.32	-69.97	2.12	2.13	0.01	#NULL!	#NULL!	#NULL!	#NULL!	#NULL!

MMP9_cha	filter_$
-4.11	0
1.20	0
11.28	1
-106.32	1
65.61	1
-10.15	0
40.98	1
-58.01	0
-30.74	0
29.96	1
-21.45	0
52.11	1
7.95	1
-26.70	0
67.46	1
5.46	0
47.99	1
-26.11	0
2.43	0
#NULL!	1
#NULL!	0
-55.91	0
44.69	1
30.43	1
-43.37	0
10.11	1
-7.02	0
#NULL!	1
37.76	1
-8.55	0
-19.30	0
#NULL!	1
#NULL!	0
#NULL!	1
58.31	1
34.34	1
-97.48	0
#NULL!	0
47.89	1
-61.40	0
69.38	1
-15.74	1
46.14	1
-0.21	0
64.98	1
-24.06	0

#NULL!	0
15.22	0
#NULL!	1
#NULL!	0
-19.11	0
62.70	1
1.86	1
#NULL!	0
-24.86	0
28.28	1
82.83	1
#NULL!	0
